# Low KIBRA Expression Is Associated with Poor Prognosis in Patients with Triple-Negative Breast Cancer

**DOI:** 10.3390/medicina57080837

**Published:** 2021-08-18

**Authors:** So-Woon Kim, Jinah Chu, Sung-Im Do, Kiyong Na

**Affiliations:** 1 Department of Pathology, Kyung Hee University Hospital, Kyung Hee University College of Medicine, 26 Kyungheedae-ro, Dongdaemun-gu, Seoul 02447, Korea; sowoonkim86@gmail.com (S.-W.K.); raripapa@gmail.com (K.N.); 2 Department of Pathology, Kangbuk Samsung Hospital, Sungkyunkwan University School of Medicine, 29 Saemunan-ro, Jongno-gu, Seoul 03181, Korea; jinah.chu@gmail.com

**Keywords:** KIBRA, breast cancer, triple-negative breast cancer, Hippo pathway

## Abstract

*Background and Objectives*: Kidney and brain protein (KIBRA) is a protein encoded by the WW and C2 domain containing 1 (*WWC1*) gene and is involved in the Hippo signaling pathway. Recent studies have revealed the prognostic value of KIBRA expression; however, its role in breast cancer remains unclear. The aim of this study was to examine KIBRA expression in relation to the clinical and pathological characteristics of patients with breast cancer and to disease outcomes. *Materials and Methods:* We analyzed the expression of KIBRA and its correlation with event-free survival (EFS) outcomes in resected samples from 486 patients with breast cancer. *Results:* KIBRA expression was significantly different among the molecular subgroups (low KIBRA expression: luminal A, 46.7% versus 50.0%, *p* = 0.641; luminal B, 32.7% versus 71.7%, *p* < 0.001; human epidermal growth factor receptor 2 (HER2)-enriched, 64.9% versus 45.5%. *p* = 0.001; triple-negative, 73.6% versus 43.8%, *p* < 0.001). Low KIBRA expression was also associated with high nuclear grade (60.4% versus 37.8%, *p* < 0.001), high histologic grade (58.7% versus 37.0%, *p* < 0.001), and estrogen receptor (ER) negativity (54.2% versus 23.6%, *p* < 0.001). Low KIBRA expression was significantly associated with poor EFS (*p* = 0.041; hazard ratio (HR) 1.658; 95% confidence interval (CI), 1.015–2.709). Low KIBRA expression was an independent indicator of poor prognosis (*p* = 0.001; HR = 3.952; 95% CI = 1.542–10.133) in triple-negative breast cancer (TNBC). *Conclusion:* Low KIBRA expression was associated with higher histological grade, ER negativity and poor EFS of breast cancer. In particular, our data highlight KIBRA expression status as a potential prognostic marker for TNBC.

## 1. Introduction

Breast cancer is the most common malignancy among women worldwide and is a leading cause of cancer-related deaths. Breast cancer accounts for 25% of all cancer cases and 15% of all cancer-related deaths [1]. Approximately 15% of breast cancers are diagnosed as triple-negative breast cancer (TNBC), and there are no approved targeted therapies for TNBC, leading to poor prognosis [2,3,4]. In this regard, concerted efforts have been made to understand the molecular basis of TNBC heterogeneity and discover actionable targets [4]. Although the TNBC subgroup is considered a single entity based on immunohistochemistry (IHC), molecular profiling has revealed an unpredictable level of heterogeneity [5]. The landmark study identified seven clusters of TNBC: basal-like 1 (BL1), basal-like 2 (BL2), immunomodulatory (IM), mesenchymal (M), mesenchymal stem-like (MSL), luminal androgen receptor (LAR), and unstable (UNS) [5,6]. Additionally, through next-generation sequencing, integrating mutation status, gene expression, and copy number, breast cancers have been segregated into ten “integrative clusters” [7]. Most TNBCs (60%) belong to integrative cluster 10 (IntClust10), with a 5-year increased risk for recurrence and frequent *TP53* mutations [1]. Up to 70% of TNBCs also have deletions on the long arm of chromosome 5, from 5q11 to 5q35 [8,9,10,11]. However, with a few exceptions, genes conferring selective pressure for 5q loss are relatively unknown [11].

A recent study by Knight et al. indicated that a gene named the WW and C2 domain containing 1 (*WWC1*) gene could inhibit breast cancer progression [1]. The *WWC1* gene located on 5q encodes a scaffold protein, the kidney and brain protein (KIBRA), which activates the Hippo pathway [12,13]. This, in turn, controls tissue growth and tumorigenesis, inhibits cell proliferation, and promotes apoptosis [14,15,16,17]. Therefore, a deletion in chromosome 5 suggests a loss of tumor suppressive function, leading to tumor proliferation, especially in TNBC [8,9,10]. Accordingly, studies have been conducted to analyze the association between KIBRA expression and prognosis in patients with breast cancer. The results revealed that KIBRA could be a potential therapeutic target for breast cancer [9,18,19].

Despite growing evidence that WWC1 is involved in breast cancer progression, few studies have evaluated the relationship between tumor characteristics and patient survival. Most studies have been laboratory investigations, indicating the need for further research using human tissues [18,19]. To address these issues, we performed clinical studies examining KIBRA expression in relation to the clinical and pathological characteristics of patients with breast cancer and disease outcomes. In particular, through an in-depth study of KIBRA expression in each subgroup of a molecular classification in patients with breast cancer, we examined whether KIBRA has potential to become a meaningful prognostic predictor.

## 2. Materials and Methods

### 2.1. Case Selection

This study (2019-05-051) was reviewed and approved by the Institutional Review Board of Kangbuk Samsung Hospital (Seoul, Korea). We searched breast cancer cases in the surgical pathology database of the Department of Pathology at Kangbuk Samsung Hospital using a combination of the keywords, “carcinoma” and “breast”. Between 2007 and 2017, 522 patients who underwent surgical resection for breast cancer were included in the study. Of the 522 cases initially evaluated for the study, 35 were excluded based on the following exclusion criteria: treatment with neoadjuvant therapy and no available clinical information. A total of 486 patients were included in this study. All cases were reviewed to verify the diagnoses; hematoxylin and eosin (HE) sections were examined by two expert pathologists (JA C and SI D) to ensure that the tissues were representative of the diagnoses. A two-step, systematic review of the routine morphological and immunohistochemical aspects of the cases was performed by the authors who were blinded to the diagnoses. Each slide was labeled with a unique study number. For the tissue microarray (TMA) analysis, two cores were acquired from each case. To minimize the possible problem of intratumoral heterogeneity, two expert pathologists (JA C and SI D) carefully selected the most representative areas morphologically for TMA by reviewing the HE slides. In addition, while reviewing the HE slides of all representative resection specimens, the relevant areas were selected as cores if there were differences in morphology, such as tumor growth pattern or differentiation.

We reviewed the medical records and pathology reports of breast cancer patients in order to document various clinicopathological parameters, including age at diagnosis, sex, histological grade, tumor size, pathological tumor stage (pT), lymph node metastasis, lymphovascular invasion, recurrence, and metastatic status at the last follow-up. Tumors were diagnosed histologically based on the World Health Organization classification, and the pathological TNM Classification of Malignant Tumors (TNM) stage was determined based on the guidelines established by the American Joint Committee on Cancer (8th edition) [20]. Histopathological review mainly focused on nuclear grade, histological subtype, and grade, including predominant growth patterns, nuclear features, and mitotic activity, which was based on the Modified Blacks nuclear grading system and Nottingham histologic score system [21,22]. To estimate event-free survival (EFS), patients were followed-up from the date of surgery to the date of death or other events, such as metastasis or recurrence. The development of local recurrence and distant metastasis was revealed on imaging analyses, including computed tomography and magnetic resonance imaging. The follow-up period for all patients was from January 2007 to December 2019.

### 2.2. Immunohistochemistry and Fluorescence In Situ Hybridization

Staining was performed using an autoimmunostainer (BenchMark XT; Ventana Medical Systems SA, Strasbourg, France) according to the manufacturer’s instructions [20]. Briefly, 4 μm-thick sections of formalin-fixed and paraffin-embedded tissue samples were deparaffinized with Bond Dewax Solution, and antigen retrieval was performed using Bond ER Solution for 30 min at 100 °C. Endogenous peroxidases were quenched by incubation with hydrogen peroxide for 5 min. The sections were incubated for 15 min at ambient temperature, with a monoclonal rabbit antibody against KIBRA (1:50, clone OTI1H8, Abcam, Cambridge, MA, USA), estrogen receptor (ER, 1:200, clone 6F11, Leica Biosystems, Buffalo Grove, IL, USA), progesterone receptor (PR, 1:200, clone 16; Leica Biosystems, Buffalo Grove, IL, USA), human epidermal growth factor receptor 2 (HER2, 1:1, clone 4B5, Ventana Medical Systems SA, Strasbourg, France), and Ki-67 (1:100, clone MIB-1, Dako, Carpinteria, CA, USA). Positive controls (normal breast parenchyma for ER and PR) and tonsil samples (KI-67 labeling index) were concurrently stained to validate the staining method. The expression status of ER, PR, HER2, and Ki-67 was evaluated according to the American Society of Clinical Oncology/College of American Pathologists guidelines (14, 15). A negative control was prepared by substituting the primary antibody with a non-immune serum sample, which resulted in no detectable staining. The equivocal staining pattern of HER2 was confirmed by fluorescence in situ hybridization using the ZytoLight SPEC HER2/CEN 17 Dual Color Probe (Zytovision, Bremerhaven, Germany). The Ki-67 labeling index was supplemented by HiPath ProTM computer-aided immunohistochemistry analysis (GenASIs, Carlsbad, CA, USA).

### 2.3. Interpretation of the KIBRA Results

**Figure 1:**
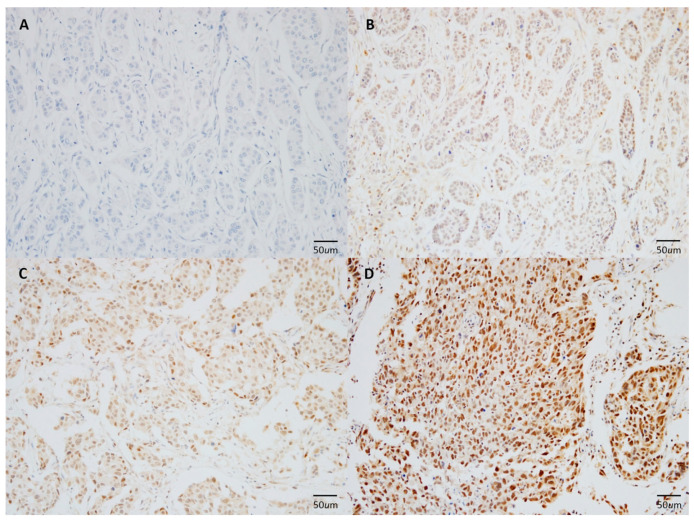
Representative immunohistochemical staining patterns of KIBRA expression in breast cancer. Shown are the representative cases with Kidney and brain protein (KIBRA)staining in increasing order: (**A**) score 0; (**B**) score 1; (**C**) score 2; (**D**) score 3. The blue color is the hematoxylin counterstain. Original magnification, ×200.

The degree of immunohistochemical KIBRA expression was semi-quantitatively determined by assessing the proportion of cancer cells that stained positively in the nucleus and the staining intensity, as previously described [18] (Figure 1). KIBRA expression was scored as follows: no staining = 0, weak staining = 1, moderate staining = 2, and strong staining = 3. The intensity of staining in normal breast epithelial cells was considered to have a score of 2, and other scores were assigned accordingly [18]. KIBRA-low was defined as an expression level in which the percentage of stained tumor cells was less than 50% and the intensity score was 0.1. All immunostained slides were examined and scored by two board-certified pathologists (S-W K and K N) who were blinded to the clinicopathological data and patient identities. The degree of agreement between the two pathologists was almost perfect (ĸ = 0.94). Disagreements between the two pathologists were resolved by consensus.

### 2.4. Statistical Analysis

The chi-squared test or Fisher’s exact test was used to examine the association between KIBRA expression status and clinicopathological parameters. Univariate survival analysis was performed to examine the prognostic significance of KIBRA expression status and clinicopathological parameters with respect to EFS. Multivariate survival analysis was performed for parameters that had a *p*-value < 0.1 in the univariate analysis using the backward stepwise elimination method with the Cox proportional hazards model (95% confidence interval). All statistical analyses were performed using IBM SPSS Statistics for Windows (version 22.0; IBM Corporation, Armonk, NY, USA). Statistical significance was set at *p* < 0.05.

## 3. Results

### 3.1. KIBRA Expression and Clinicopathological Characteristics

A total of 486 patients were included in the analysis. Briefly, the median age was 50 years (range 28–87 years) and the median follow-up time was 75.5 months. The histopathological subtypes of breast cancer were invasive breast carcinoma of no special type (IBC-NST; *n* = 423), invasive lobular carcinoma (ILC; *n* = 13), medullary carcinoma (*n* = 19), mucinous carcinoma (*n* = 17), metaplastic carcinoma (*n* = 13), and pleomorphic ILC (*n* = 1). Of the 486 patients, 238 (49.0%) and 232 (47.7%) had high nuclear and histologic grades, respectively. Additionally, 257 patients (50.0%) had lymph node metastasis and 317 patients (65.2%) had lymphovascular invasion.

The cases were divided into two groups according to the total immunostaining score: low-expression and high-expression. The low-expression group included 240 patients (49.3%). Table 1 summarizes the association between KIBRA expression status and the clinicopathological parameters of breast cancer. Low KIBRA expression was also associated with high nuclear grade (60.4% versus 37.8%, *p* < 0.001), high histologic grade (58.7% versus 37.0%, *p* < 0.001), and ER negativity (54.2% versus 23.6%, *p* < 0.001). In the molecular subtypes, KIBRA expression was significantly different among each subgroup, except luminal A (low KIBRA expression: luminal A, 46.7% versus 50.0%, *p* = 0.641; luminal B, 32.7% versus 71.7%, *p* < 0.001; HER2-enriched, 64.9% versus 45.5%, *p* = 0.001; triple-negative, 73.6% versus 43.8%, *p* < 0.001). Low expression of KIBRA was significantly increased in HER2-enriched breast cancer and TNBC, which corroborates its association with ER negativity. No significant differences in age, pathological tumor stage, lymph node metastasis, lymphatic invasion, and HER2-status were noted between the KIBRA-high and KIBRA-low groups.

### 3.2. Clinicopathological Significance of KIBRA Expression in Breast Cancer According to Molecular Subtypes

The cohort was divided into four molecular subtypes: luminal A (*n* = 90, 2.7%), luminal B (*n* = 208, 2.7%), HER2-enriched (*n* = 97, 2.7%), and TNBC (*n* = 91, 2.7%). Next, we re-analyzed the relationship between KIBRA expression status and the clinicopathological parameters of breast cancer within each molecular subtype (Table 2). In the luminal A and HER2-enriched subtypes, older patients had significantly lower KIBRA expression than younger patients (mean ± standard deviation: luminal A, 52 ± 10.08 versus 48 ± 8.5, *p* = 0.003; HER2-enriched, 54 ± 11.36 versus 48 ± 14.48, *p* = 0.044). In the luminal B and HER2-enriched subtypes, low KIBRA expression was associated with high nuclear grade (luminal B, 60.3% versus 39.7%, *p* = 0.003; HER2-enriched, 81.0% versus 19.0%, *p* = 0.03) and high histologic grade (luminal B, 64.7% versus 35.3%, *p* = 0.002; HER2-enriched, 77.8% versus 22.2%, *p* = 0.007). No significant differences were noted in the TNBC subtype.

### 3.3. Prognostic Implication of KIBRA Expression in Breast Cancer

The median follow-up, as assessed by the Kaplan–Meier method, was 75.5 months; 69 of the 486 patients (14%) had recurrence or metastasis during follow-up. Of the 486 patients, 240 (49.4%) were classified as KIBRA-low; their median EFS was 56 months versus 74 months for the 246 patients in the KIBRA-high group. Low expression of KIBRA was significantly associated with poor EFS (*p* = 0.041, HR = 1.658, 95% confidence interval [CI] = 1.015–2.709), when compared to the EFS of KIBRA-high patients (Table 3). Additionally, the EFS rate at 5 years was 58.8% in the KIBRA-low group and 51.6% in the KIBRA-high group.

**Figure 2:**
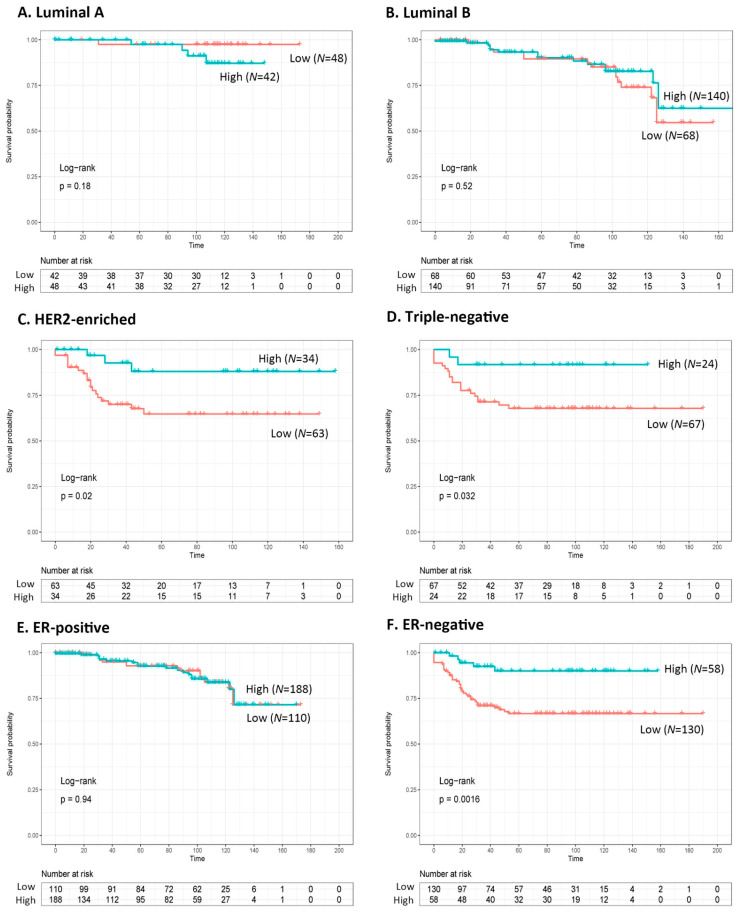
Kaplan–Meier curves generated in accordance with the KIBRA expression in patients with breast cancer (**A**,**B**). Patients in luminal A and B subtypes have no significant difference in terms of survival between low- and high-KIBRA expression (*p* = 0.18 and *p* = 0.052, respectively). (**C**,**D**) Patients with low KIBRA expression showed significantly poorer event-free survival than patients with high KIBRA expression in HER2-enriched and triple-negative subtypes (*p* = 0.02 and *p* = 0.032, respectively). (**E**,**F**) After dichotomization for ER status, patients with low KIBRA expression show significantly poorer event-free survival than patients with high KIBRA expression only in the ER-negative group (*p* = 0.0016).

The correlation between EFS and molecular subtypes was similar to that observed in previous reports [7]; luminal A tumors showed the best prognoses, followed by the luminal B, HER2-enriched, and TNBC subtypes, which showed the worst prognoses (*p* < 0.001). Notably, the expression status of KIBRA differed significantly according to the molecular subtype (Figure 2). Compared to the luminal type (Figure 2A,B), low KIBRA expression was associated with significantly worse EFS in the HER2-enriched and triple-negative breast cancer subtypes (Figure 2C,D). Together, they were divided into two groups according to ER status; low KIBRA expression had a significantly worse EFS in ER-negative breast cancer (Figure 2E,F).

### 3.4. KIBRA Expression Is an Independent Prognostic Factor for TNBC

Significant benefits concerning EFS were observed in patients with ER-negative breast cancer in the KIBRA-high group. Cox regression analysis was performed for other clinicopathological factors to determine whether KIBRA expression was an independent prognostic factor (Table 3). Univariate analysis showed that KIBRA expression, lymph node metastasis, and lymphovascular invasion were adverse prognostic factors for EFS. Factors found to be significant in the univariate analysis were included in the multivariate analysis. After adjusting for these variables, KIBRA expression and lymph node metastasis were found to be independent prognostic factors for EFS (KIBRA, *p* = 0.004, HR = 3.952, 95% CI = 1.542–10.133; lymph node metastasis, *p* < 0.001, HR = 6.597, 95% CI, 2.786–15.623) (Table 3).

## 4. Discussion

In this study, we aimed to investigate whether the expression status of KIBRA has prognostic implications and clinicopathological significance in breast cancer. In our study, low expression of KIBRA was frequently detected in ER-negative patients, corroborating previous studies showing that KIBRA plays a role in ER transactivation in breast cancer cells (Table 1) [19]. KIBRA directly binds to dynein light chain 1 (DLC1), an ER-interacting protein, and acts as a downstream mediator of the regulation of ER transactivation by DLC1 [23]. Since KIBRA maintains the functionality of the ER, a relationship must exist between the expression level of KIBRA and ER status.

We show here that low-KIBRA expression is an independent poor prognostic factor in TNBC, regardless of adjuvant treatment. Previously, Wang et al. demonstrated that KIBRA was significantly associated with advanced-stage diseases, high-grade tumors, and ER- or PR-negative status [19]. Mudduwa et al. demonstrated that KIBRA has an independent effect on the recurrence-free survival of luminal breast cancer patients who received limited adjuvant endocrine therapy and chemotherapy [18]. Although the results of the present and previous studies showed different prognoses according to the molecular subtype of breast cancer, this may be attributed to different interpretations of the KIBRA expression site and of the proportion of stained cancer cells. The results of the present study are consistent with the notion that low-KIBRA expression plays an important role in the prognosis of patients with breast cancer. Based on our results, we suggest that in the case of TNBC, caution is needed while interpreting KIBRA expression considering the location and proportion of tumors.

Of note, in our study, low KIBRA expression was also significantly associated with high-grade morphologic features, which is consistent with the characteristics of patients with ER-negative breast cancer, especially TNBC. Much of the premise for KIBRA as a tumor suppressor comes from its role in activating the Hippo signaling pathway, for which loss of function and the concomitant activation of YAP/TAZ are well-documented in TNBCs [24]. The role of KIBRA in suppressing mechanical signals activating TAZ may be related to the suppression of self-renewal, given that an undifferentiated stem-like state is maintained through contact with the extracellular matrix [25,26]. Indeed, cells maintaining extracellular matrix contact in the basal layer of breast epithelium have nuclear TAZ, which becomes cytoplasmic as cells differentiate and lose basement membrane contact [27]. KIBRA loss may constitutively activate mechanotransduction pathways that positively regulate TAZ, leading to persistent TAZ nuclear localization and the maintenance of the poorly differentiated phenotype associated with basal-like tumors [27]. In short, there seems to be a significant correlation between the expression of KIBRA and ER status, which leads to a morphological correlation likely affecting the poor prognosis of KIBRA.

We found a prognostic effect of KIBRA in patients with breast cancer, especially in patients with ER-negative cancer containing the HER2-enriched and TBNC subtypes. These results can be verified by the fact that KIBRA plays an important role in the Hippo signaling pathway, which functions differently depending on the molecular subtype of breast cancer [28,29,30,31]. The Hippo signaling pathway is a pathway that has recently been said to have several oncogenic functions in breast cancer [28]. Hippo molecular elements—KIBRA, YAP/TAZ, Aurora kinase, and LATS—have been shown to participate in breast cancer development through different mechanisms, resulting in different roles among its molecular subtypes [24]. In particular, among molecular subtypes, chromosome 5q loss is detected in 70% of TNBC, which involves the Hippo molecular element, KIBRA, a major factor contributing to its effects on tumor growth and metastatic progression. Mechanistically, KIBRA suppresses RhoA activation, impairing the nuclear translocation of the oncogenes, and YAP/TAZ, which drives metastatic and cancer stem-cell-like behavior [24]. Through this series of processes, it has been suggested that the loss of multiple DNA damage response and cell cycle genes upon 5q deletion may promote genomic instability and tumor progression [7,32]. To address this issue, we found a significant association between KIBRA expression status and EFS rate in TNBC, which means that patients with low KIBRA expression in TNBC are more likely to metastasize or recur, demonstrating the contributing metastatic nature of KIBRA.

In general, KIBRA is expressed in normal breast tissue at all stages of glandular development. Strong nuclear expression of KIBRA is maintained in lactating mammary acini, with an ability to increase the proliferation of mammary acinar cells [33]. In breast cancer, KIBRA has been recently reported to be expressed in both the nucleus and cytoplasm of breast cancer cells, and its prognostic value depends on its nuclear expression [18]. In line with our study, loss of KIBRA expression at the nuclear level was a significantly poor prognostic factor. As a tumor suppressor gene, nuclear localization of KIBRA has functional significance in deregulating cell division and the cell cycle, suggesting that low KIBRA expression with nuclear localization affects the aggressiveness of breast cancer [24,34,35]. In fact, KIBRA cooperatively functions with the protein tyrosine phosphatase, PTPN14, to trigger mechanotransduction-regulated signals that inhibit the nuclear localization of oncogenic transcriptional co-activators, YAP/TAZ [24].

The current study has several limitations, including its retrospective single-center design, semiquantitative assessment of KIBRA expression, exclusion of patients who received neoadjuvant chemotherapy, and the inability to evaluate the relationship between KIBRA expression and overall patient survival. Furthermore, the mechanisms underlying KIBRA in HER2-enriched-subtype carcinogenesis have not been examined. Future studies on breast cancer should include external validation, and additional experimental studies are also needed.

## 5. Conclusions

In conclusion, our results indicate that low expression of KIBRA in TNBC is independently and strongly related to poor prognosis. These results suggest that the KIBRA marker can be used as a prognostic marker in patients at risk of an aggressive disease process in TNBC.

## Figures and Tables

**Table 1:** Relationship between KIBRA expression status and the clinicopathological parameters of breast cancer patients.

Parameter	Number of Cases	KIBRA Expression		*p*-Value
		Low	High	
		(*n* = 240)	(*n* = 246)	
		*n* (%)	*n* (%)	
Age (years), mean ± standard deviation	486	52 ± 11.16	50 ± 11.34	0.213
Nuclear grade				**<0.001**
Low (1–2)	248	95 (39.6)	153 (62.2)	
High (3)	238	145 (60.4)	93 (37.8)	
Histologic grade				**<0.001**
Low (1–2)	254	99 (41.3)	155 (63.0)	
High (3)	232	141 (58.7)	91 (37.0)	
Pathological tumor stage (pT)				0.069
1–2	223	100 (41.7)	123 (50.0)	
3–4	263	140 (58.3)	123 (50.0)	
Lymph node metastasis				1
Absent	257	127 (52.9)	130 (52.8)	
Present	229	113 (47.1)	116 (47.2)	
Lymphovascular invasion				0.924
Absent	317	156 (65.0)	161 (65.4)	
Present	169	84 (35.0)	85 (34.6)	
Estrogen receptor				**<0.001**
Negative	188	130 (54.2)	58 (23.6)	
Positive	298	110 (45.8)	188 (76.4)	
HER2				0.308
Negative	192	89 (37.1)	103 (41.9)	
Positive	294	151 (62.9)	143 (58.1)	
Molecular subtype				**<0.001**
Luminal A	90	42 (17.5)	48 (19.5)	
Luminal B	208	68 (28.3)	140 (56.9)	
HER2-enriched	97	63 (26.3)	34 (13.8)	
Triple-negative	91	67 (27.9)	24 (9.8)	

KIBRA, Kidney and brain protein; HER2, human epidermal growth factor receptor 2; Bold values indicate statistically significant values (*p* < 0.05).

**Table 2:** Relationship between KIBRA expression status and the clinicopathological parameters of breast cancer patients according to the molecular subgroups.

KIBRA	Luminal A		*p*-Value	Luminal B		*p*-Value	HER2-Enriched		*p*-Value	Triple-Negative		*p*-Value
	Low	High		Low	High		Low	High		Low	High	
	(*n* = 42)	(*n* = 48)		(*n* = 68)	(*n* = 140)		(*n* = 63)	(*n* = 34)		(*n* = 67)	(*n* = 24)	
	*n* (%)	*n* (%)		*n* (%)	*n* (%)		*n* (%)	*n* (%)		*n* (%)	*n* (%)	
Age (years), mean ± standard deviation	52 ± 10.08	48 ± 8.5	0.036	49 ± 11.64	52 ± 11.30	0.148	54 ± 11.36	48 ± 14.48	**0.044**	51 ± 10.87	49 ± 10.80	0.33
Nuclear grade			1			**0.003**			**0.03**			0.602
Low (1–2)	38 (90.5)	44 (91.7)		27 (39.7)	87 (62.1)		12 (19.0)	14 (41.2)		18 (26.9)	8 (33.3)	
High (3)	4 (9.5)	4 (8.3)		41 (60.3)	53 (37.9)		51 (81.0)	20 (58.8)		49 (73.1)	16 (66.7)	
Histologic grade			1			**0.002**			**0.007**			0.319
Low (1–2)	41 (97.6)	46 (95.8)		24 (35.3)	82 (58.6)		14 (22.2)	17 (50.0)		20 (29.9)	10 (41.7)	
High (3)	1 (2.4)	2 (4.2)		44 (64.7)	58 (41.4)		49 (77.8)	17 (50.0)		47 (70.1)	14 (58.3)	
Pathological tumor stage (pT)			0.828			0.369			0.091			0.324
1–2	27 (64.3)	32 (66.7)		24 (35.3)	59 (42.1)		27 (42.9)	21 (61.8)		22 (32.8)	11 (45.8)	
3–4	15 (35.7)	16 (33.3)		44 (64.7)	81 (57.9)		36 (57.1)	13 (38.2)		45 (67.2)	13 (54.2)	
Lymph node metastasis			1			0.768			0.672			1
Absent	23 (54.8)	27 (56.3)		37 (54.4)	72 (51.4)		28 (44.4)	17 (50.0)		39 (58.2)	14 (58.3)	
Present	19 (45.2)	21 (43.8)		31 (45.6)	68 (48.6)		35 (55.6)	17 (50.0)		28 (41.8)	10 (41.7)	
Lymphovascular invasion			1			0.23			0.085			0.194
Absent	33 (78.6)	37 (77.1)		45 (66.2)	80 (57.1)		32 (50.8)	24 (70.6)		46 (68.7)	20 (83.3)	
Present	9 (21.4)	11 (22.9)		23 (33.8)	60 (42.9)		31 (49.2)	10 (29.4)		21 (31.3)	4 (16.7)	

Bold values indicate statistically significant values (*p* < 0.05).

**Table 3:** Event-free survival analyses of patients with triple-negative breast cancer.

Factor	Univariate Analysis			Multivariate Analysis		
	HR	95% CI	*p*	HR	95% CI	*p*
KIBRA			**0.001**			**0.004**
High	1			1		
Low	3.958	1.562–10.03		3.952	1.542–10.133	
Lymph node metastasis			**<0.001**			**<0.001**
Absent	1			1		
Present	7.561	3.37–16.965		6.597	2.786–15.623	
Lymphovascular invasion			**<0.001**			0.198
Absent	1			1		
Present	3.629	1.983–6.640		1.532	0.800–2.931	
Age, years			0.557			
<60	1					
≥60	1.226	0.643–2.422				
Pathological tumor stage (pT)			0.242			
1–2	1					
3–4	1.44	0.782–2.651				
Nuclear grade			0.969			
Low (1–2)	1					
High (3)	1.006	0.729–1.389				
Histologic grade			0.494			
Low (1–2)	1					
High (3)	1.252	0.647–2.386				

HR, hazard ratio; CI, confidence interval. Bold values indicate statistically significant values (*p* < 0.05).

## Data Availability

The data presented in this study are available on request from the corresponding author. The data are not publicly available due to privacy and ethical restrictions.

## References

[1] Knight J.F., Sung V.Y.C., Kuzmin E., Couzens A.L., de Verteuil D.A., Ratcliffe C.D.H., Coelho P.P., Johnson R.M., Samavarchi-Tehrani P., Gruosso T. (2018). KIBRA (WWC1) Is a Metastasis Suppressor Gene Affected by Chromosome 5q Loss in Triple-Negative Breast Cancer. Cell Rep..

[2] Foulkes W.D., Smith I.E., Reis-Filho J.S. (2010). Triple-negative breast cancer. N. Engl. J. Med..

[3] Bianchini G., Balko J.M., Mayer I.A., Sanders M.E., Gianni L. (2016). Triple-negative breast cancer: Challenges and opportunities of a heterogeneous disease. Nat. Rev. Clin. Oncol..

[4] Denkert C., Liedtke C., Tutt A., von Minckwitz G. (2017). Molecular alterations in triple-negative breast cancer-the road to new treatment strategies. Lancet.

[5] Marra A., Trapani D., Viale G., Criscitiello C., Curigliano G. (2020). Practical classification of triple-negative breast cancer: Intratumoral heterogeneity, mechanisms of drug resistance, and novel therapies. NPJ Breast Cancer.

[6] Lehmann B.D., Jovanović B., Chen X., Estrada M.V., Johnson K.N., Shyr Y., Moses H.L., Sanders M.E., Pietenpol J.A. (2016). Refinement of Triple-Negative Breast Cancer Molecular Subtypes: Implications for Neoadjuvant Chemotherapy Selection. PLoS ONE.

[7] Curtis C., Shah S.P., Chin S.F., Turashvili G., Rueda O.M., Dunning M.J., Speed D., Lynch A.G., Samarajiwa S., Yuan Y. (2012). The genomic and transcriptomic architecture of 2000 breast tumours reveals novel subgroups. Nature.

[8] Johannsdottir H.K., Jonsson G., Johannesdottir G., Agnarsson B.A., Eerola H., Arason A., Heikkila P., Egilsson V., Olsson H., Johannsson O.T. (2006). Chromosome 5 imbalance mapping in breast tumors from BRCA1 and BRCA2 mutation carriers and sporadic breast tumors. Int. J. Cancer.

[9] Natrajan R., Lambros M.B., Rodríguez-Pinilla S.M., Moreno-Bueno G., Tan D.S., Marchió C., Vatcheva R., Rayter S., Mahler-Araujo B., Fulford L.G. (2009). Tiling path genomic profiling of grade 3 invasive ductal breast cancers. Clin. Cancer Res..

[10] Turner N., Lambros M.B., Horlings H.M., Pearson A., Sharpe R., Natrajan R., Geyer F.C., van Kouwenhove M., Kreike B., Mackay A. (2010). Integrative molecular profiling of triple negative breast cancers identifies amplicon drivers and potential therapeutic targets. Oncogene.

[11] Wilson K.E., Li Y.W., Yang N., Shen H., Orillion A.R., Zhang J. (2014). PTPN14 forms a complex with Kibra and LATS1 proteins and negatively regulates the YAP oncogenic function. J. Biol. Chem..

[12] Baumgartner R., Poernbacher I., Buser N., Hafen E., Stocker H. (2010). The WW domain protein Kibra acts upstream of Hippo in Drosophila. Dev. Cell.

[13] Yu J., Zheng Y., Dong J., Klusza S., Deng W.M., Pan D. (2010). Kibra functions as a tumor suppressor protein that regulates Hippo signaling in conjunction with Merlin and Expanded. Dev. Cell.

[14] Edgar B.A. (2006). From cell structure to transcription: Hippo forges a new path. Cell.

[15] Harvey K., Tapon N. (2007). The Salvador-Warts-Hippo pathway—An emerging tumour-suppressor network. Nat. Rev. Cancer.

[16] Harvey K.F., Pfleger C.M., Hariharan I.K. (2003). The Drosophila Mst ortholog, hippo, restricts growth and cell proliferation and promotes apoptosis. Cell.

[17] Huang J., Wu S., Barrera J., Matthews K., Pan D. (2005). The Hippo signaling pathway coordinately regulates cell proliferation and apoptosis by inactivating Yorkie, the Drosophila Homolog of YAP. Cell.

[18] Mudduwa L., Peiris H., Gunasekara S., Abeysiriwardhana D., Liyanage N., Rayala S.K., Liyanage T. (2018). KIBRA; a novel biomarker predicting recurrence free survival of breast cancer patients receiving adjuvant therapy. BMC Cancer.

[19] Wang Z., Katsaros D., Biglia N., Shen Y., Fu Y., Tiirikainen M., Yu H. (2019). Low expression of WWC1, a tumor suppressor gene, is associated with aggressive breast cancer and poor survival outcome. FEBS Open Bio.

[20] Kim S.W., Kim H.S., Na K. (2020). Characterization of Paired Box 8 (PAX8)-expressing Metastatic Breast Carcinoma. Anticancer Res..

[21] Elston C.W., Ellis I.O. (1991). Pathological prognostic factors in breast cancer. I. The value of histological grade in breast cancer: Experience from a large study with long-term follow-up. Histopathology.

[22] Schuh F., Biazús J.V., Resetkova E., Benfica C.Z., Ventura A.d.F., Uchoa D., Graudenz M., Edelweiss M.I.A. (2015). Histopathological grading of breast ductal carcinoma in situ: Validation of a web-based survey through intra-observer reproducibility analysis. Diagn. Pathol..

[23] Rayala S.K., den Hollander P., Manavathi B., Talukder A.H., Song C., Peng S., Barnekow A., Kremerskothen J., Kumar R. (2006). Essential role of KIBRA in co-activator function of dynein light chain 1 in mammalian cells. J. Biol. Chem..

[24] Kyriazoglou A., Liontos M., Zakopoulou R., Kaparelou M., Tsiara A., Papatheodoridi A.M., Georgakopoulou R., Zagouri F. (2021). The Role of the Hippo Pathway in Breast Cancer Carcinogenesis, Prognosis, and Treatment: A Systematic Review. Breast Care.

[25] Engler A.J., Sen S., Sweeney H.L., Discher D.E. (2006). Matrix elasticity directs stem cell lineage specification. Cell.

[26] Lui C., Lee K., Nelson C.M. (2012). Matrix compliance and RhoA direct the differentiation of mammary progenitor cells. Biomech. Model. Mechanobiol..

[27] Skibinski A., Breindel J.L., Prat A., Galván P., Smith E., Rolfs A., Gupta P.B., LaBaer J., Kuperwasser C. (2014). The Hippo transducer TAZ interacts with the SWI/SNF complex to regulate breast epithelial lineage commitment. Cell Rep..

[28] Maugeri-Saccà M., Barba M., Pizzuti L., Vici P., Di Lauro L., Dattilo R., Vitale I., Bartucci M., Mottolese M., De Maria R. (2015). The Hippo transducers TAZ and YAP in breast cancer: Oncogenic activities and clinical implications. Expert Rev. Mol. Med..

[29] Bendinelli P., Maroni P., Matteucci E., Luzzati A., Perrucchini G., Desiderio M.A. (2013). Hypoxia inducible factor-1 is activated by transcriptional co-activator with PDZ-binding motif (TAZ) versus WWdomain-containing oxidoreductase (WWOX) in hypoxic microenvironment of bone metastasis from breast cancer. Eur. J. Cancer.

[30] Visser S., Yang X. (2010). LATS tumor suppressor: A new governor of cellular homeostasis. Cell Cycle.

[31] Lehn S., Tobin N.P., Sims A.H., Stål O., Jirström K., Axelson H., Landberg G. (2014). Decreased expression of Yes-associated protein is associated with outcome in the luminal A breast cancer subgroup and with an impaired tamoxifen response. BMC Cancer.

[32] Weigman V.J., Chao H.H., Shabalin A.A., He X., Parker J.S., Nordgard S.H., Grushko T., Huo D., Nwachukwu C., Nobel A. (2012). Basal-like Breast cancer DNA copy number losses identify genes involved in genomic instability, response to therapy, and patient survival. Breast Cancer Res. Treat..

[33] Hilton H.N., Stanford P.M., Harris J., Oakes S.R., Kaplan W., Daly R.J., Ormandy C.J. (2008). KIBRA interacts with discoidin domain receptor 1 to modulate collagen-induced signalling. Biochim. Biophys. Acta.

[34] Zanconato F., Battilana G., Cordenonsi M., Piccolo S. (2016). YAP/TAZ as therapeutic targets in cancer. Curr. Opin. Pharmacol..

[35] Ji M., Yang S., Chen Y., Xiao L., Zhang L., Dong J. (2012). Phospho-regulation of KIBRA by CDK1 and CDC14 phosphatase controls cell-cycle progression. Biochem. J..

